# Albumin administration in the acutely ill: what is new and where next?

**DOI:** 10.1186/cc13991

**Published:** 2014-07-16

**Authors:** Jean-Louis Vincent, James A Russell, Matthias Jacob, Greg Martin, Bertrand Guidet, Jan Wernerman, Ricard Ferrer Roca, Stuart A McCluskey, Luciano Gattinoni

**Affiliations:** 1 Department of Intensive Care, Erasme Hospital, Université libre de Bruxelles, route de Lennik 808, 1070 Brussels, Belgium; 2 Center for Heart Lung Innovation and Critical Care Medicine, University of British Columbia and St. Paul's Hospital, Vancouver, Canada V6Z BC; 3 Department of Anaesthesiology, University Hospital Munich, Nussbaumstraße 20, 80336 Munich, Germany; 4 Department of Medicine, Division of Pulmonary, Allergy and Critical Care, Emory University School of Medicine, Atlanta, GA 30303, USA; 5 Service Réanimation Médicale, Hôpital Saint Antoine, Universitaires Est Parisien, Paris, France; 6 Sorbonne Universités, UPMC Université Paris 06, UMR-S 1136, Institut Pierre Louis d’Epidémiologie et de Santé Publique, F-75013 Paris, France; 7 Department of Anesthesiology & Intensive Care Medicine, Karolinska University Hospital, Huddinge, 14186 Stockholm, Sweden; 8 Servei de Medicina Intensiva, Hospital Universitari Mútua Terrassa, Universitat de Barcelona, CIBER Enfermedades Respiratorias, 08221 Terrassa, Barcelona, Spain; 9 Department of Anesthesia and Pain Management, Toronto General Hospital, University of Toronto, Toronto, Ontario, Canada M5G 2C4; 10 Dipartimento di Anestesia, Rianimazione (Intensiva e Subintensiva) e Terapia del Dolore, Fondazione IRCCS Ca' Granda – Ospedale Maggiore Policlinico, 20122 Milan, Italy

## Abstract

Albumin solutions have been used worldwide for the treatment of critically ill patients since they became commercially available in the 1940s. However, their use has become the subject of criticism and debate in more recent years. Importantly, all fluid solutions have potential benefits and drawbacks. Large multicenter randomized studies have provided valuable data regarding the safety of albumin solutions, and have begun to clarify which groups of patients are most likely to benefit from their use. However, many questions remain related to where exactly albumin fits within our fluid choices. Here, we briefly summarize some of the physiology and history of albumin use in intensive care before offering some evidence-based guidance for albumin use in critically ill patients.

## Introduction

Albumen is doubtless one of the most important of the animal proximate principles.

### (Henry Ancell [1])

Albumin solutions have been used worldwide for the treatment of critically ill patients since they became commercially available in the 1940s. However, driven largely by the results of a widely publicized meta-analysis in 1998 that reported increased mortality rates in patients who received albumin solutions [2], the role of albumin administration in critically ill patients became highly controversial. It is well known that albumin has multiple physiological effects [3], including regulation of colloid osmotic pressure (COP), binding and transportation of various substances (for example, drugs, hormones) within the blood, antioxidant properties, nitric oxide modulation and buffer capabilities, which may be of particular relevance in critically ill patients. It is also well established that low serum albumin levels, a common occurrence in critically ill patients, are associated with worse outcomes [4,5]. There would therefore seem to be a good rationale for use of albumin infusions in critically ill patients. However, albumin solutions also have limitations, including high costs relative to possible alternatives, notably crystalloids, and potential (rare) risks of transmission of microorganisms, anticoagulant, and allergic effects [6-8]. Because there are no definitive randomized controlled trials (RCTs) demonstrating an outcome benefit of albumin in heterogeneous groups of critically ill patients, routine administration of albumin for fluid resuscitation is not warranted in all patients, but there is evidence to support its use in some patient populations.

The purpose of this article is not to review in detail the multiple functions and roles of albumin or the many comparative studies and meta-analyses that have now been performed, although we will briefly summarize this information to provide some context. Rather, we wish to provide some clear suggestions and guidance for albumin use based on the current available evidence and highlight important areas for future research.

## Some background

### History

Albumin was one of the first human proteins to be isolated and extracted from plasma for clinical use. First crystallized in 1934, a preparation was made available for clinical use in the 1940s [9,10]. Early successful use in multi-trauma and severely burned patients led to rapid expansion of the so-called human albumin program in the USA [11], and albumin use spread from the military setting to civilian hospitals and into regular use in operating and emergency rooms around the world. The first commercially available preparations of intravenous human albumin solution were developed using the cold alcohol fractionation technique created by Edwin Joseph Cohn [9,11]. Later developments and refinements in extraction and processing have resulted in increasingly pure solutions [12].

### Physiological properties

**Figure 1:**
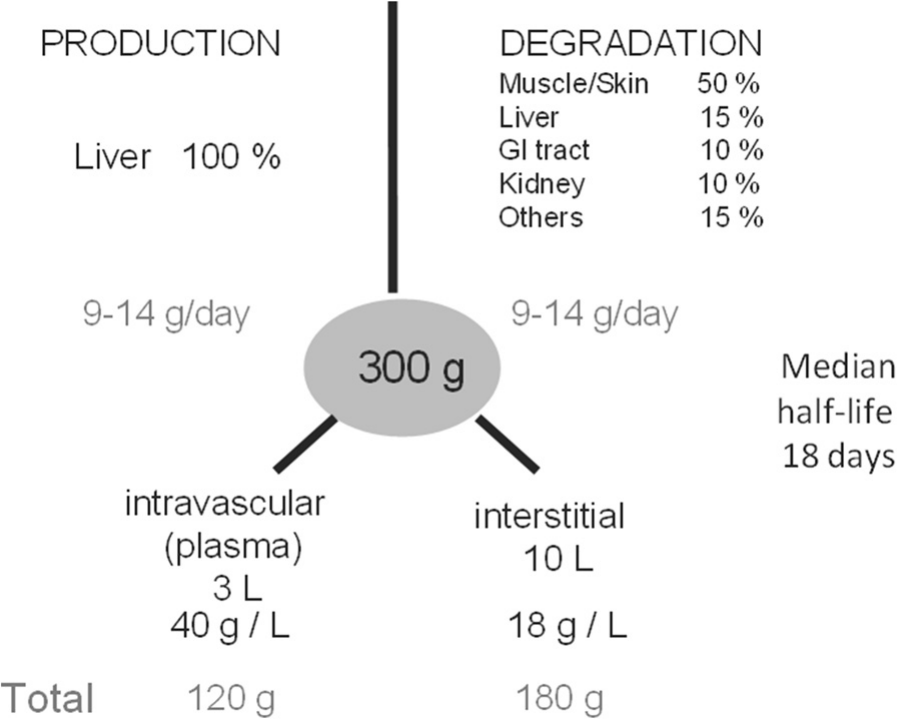
**Schematic illustration of metabolism of albumin in healthy adults.** GI, gastrointestinal.

Albumin is a natural plasma protein synthesized exclusively by the liver at a rate of 9 to 14 g/day in healthy individuals, with a median half-life of about 18 to 19 days (Figure 1) [9]. Albumin is catabolized in most organs of the body at a similar rate of about 9 to 14 g/day, by uptake into endocytotic vesicles on the endothelial surface [9,13]; the final breakdown products are amino acids [13].

**Figure 2:**
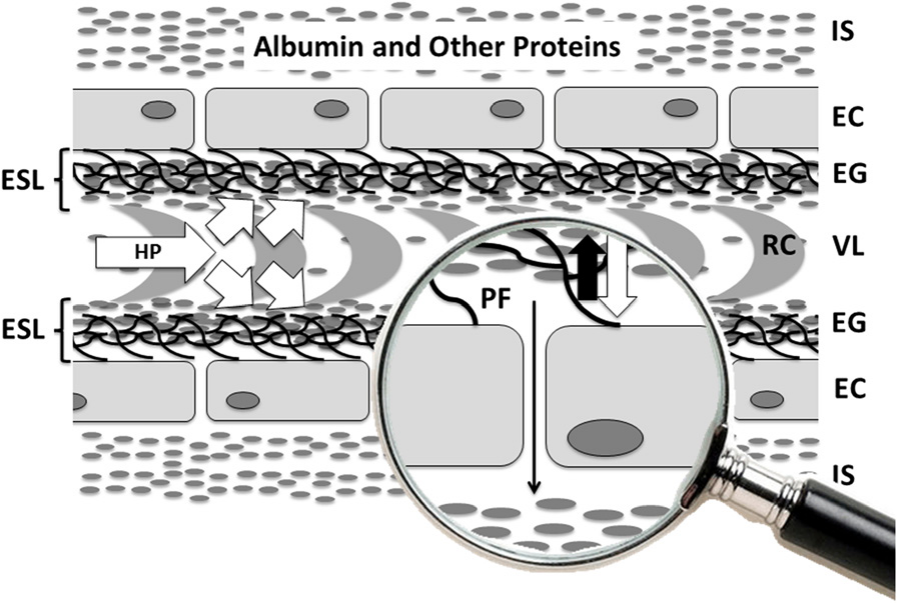
**Schematic illustration of the current understanding of vascular barrier function within the high-pressure segment of the vascular system.** For explanation, see text. White arrows, hydrostatic pressure (HP) gradients towards the interstitial space; thick black arrow, inward directed oncotic force across the endothelial surface layer; thin black arrow, small flux of protein low ultrafiltrate. EC, endothelial cell; EG, endothelial glycocalyx; ESL, endothelial surface layer; IS, interstitial space; PF, protein free space beneath the endothelial surface layer; RC, red blood cell; VL, vascular lumen.

Albumin has well-known effects on maintaining fluid balance, being responsible for 75 to 80% of COP in the basal physiological state [9,10]. In critically ill patients, particularly those with sepsis, the relationship between COP and the albumin concentration is complex, being influenced by altered permeability and increased transcapillary escape rates [14,15]. Moreover, improved understanding of the endothelial glyocalyx has altered our comprehension of the role of COP in fluid balance [16]. Numerous experimental studies have confirmed that the traditional understanding of an inwards-directed oncotic gradient between a protein-low interstitial space and a protein-rich plasma, as suggested by Ernest Starling more than 100 years ago, is not correct; indeed, the interstitial compartment has high protein concentrations. Nevertheless, there is a functional vascular barrier, created by the endothelial glycocalyx layer, a skeleton of glycoproteins, proteoglycans and glycosaminoglycans, and its interaction with plasma constituents, including albumin, to form the endothelial surface layer, which is positioned on the luminal side of the endothelium with a thickness of up to 1 μm in humans [16-19]. A small space, situated on the luminal side of the endothelium beneath this protein sponge, is permanently cleared of passing protein molecules by a protein-low resting flux through small breaks in the intercellular junction strands towards the tissues [18]. Accordingly, an inwardly-directed oncotic force, quantitatively opposite to the hydrostatically driven fluid filtration, develops exclusively across the small space beneath the glycocalyx and the protein-loaded endothelial surface layer (Figure 2).

Current understanding of optimal vascular barrier function in the high-pressure segment of the vascular system includes an intact glycocalyx combined with a minimum concentration of plasma proteins [20]. Although albumin is therefore a crucial part of the endothelial surface layer and infusing albumin may seem a reasonable suggestion to improve and maintain vascular barrier competence, experiments in isolated organs have shown that the endothelial surface layer appears to function well until the albumin concentration falls to a value as low as around 10 g/l [21]. Hence, the major insult when the vascular barrier fails to function because of a severe acute illness is most probably not hypoalbuminemia, but a breakdown of the molecular structure of the endothelial glycocalyx because of hypervolemia or ischemia/reperfusion injury and other forms of systemic inflammation [22]. Nevertheless, below a certain threshold, artificial substitutes such as starches or gelatin are not sufficient to form an endothelial surface layer with a resistance against pressure-dependent fluid and protein outflow comparable with albumin [21,23].

Albumin has many other properties in addition to its effects on intravascular volume, including transport and antioxidant activities, but their importance in health and disease are less well documented.

The antioxidant effects of albumin are, in brief, related to its ability to bind certain ligands, notably iron and copper, which reduces the availability of these compounds for pro-oxidant reactions, and are related to an exposed thiol group on the free cysteine residue, which acts as a free radical scavenger, able to interact with or trap reactive oxygen or nitrogen species, including nitric oxide, a key mediator in many conditions including sepsis [10,24-26].

**Figure 3:**
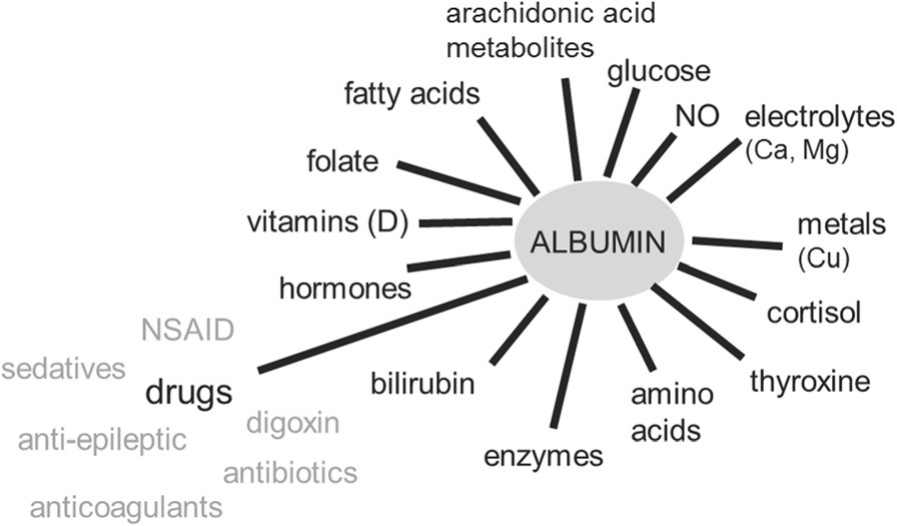
**Some of the key substances transported by albumin.** NO, nitric oxide; NSAID, nonsteroidal anti-inflammatory drug.

In addition to the binding of iron and copper ions, albumin also transports multiple other endogenous and exogenous substances (Figure 3) [13]. Changes in albumin concentrations and structure during critical illness can therefore potentially have marked effects on normal homeostasis and metabolism and on drug delivery and efficacy [10,27]. In a systematic review, Ulldemolins and colleagues reported that protein binding of antibacterials, including ceftriaxone, ertapenem, teicoplanin, and aztreonam, was frequently decreased in critically ill patients with hypoalbuminemia, notably with increased volume of distribution and drug clearance [27]. These changes could result in suboptimal treatment, particularly for time-dependent antibiotics, and may necessitate dose adjustment.

The balance of acidic to basic residues on albumin makes it a weak acid in physiological concentrations [10,28], so that a decrease in albumin concentration increases the anion gap. This passively increases bicarbonate concentration, and is therefore associated with development of metabolic alkalosis.

Albumin also has anticoagulant effects similar to, but much less potent than, those of heparin, and inhibits platelet aggregation [29].

Finally, albumin can protect the microvasculature and mitigate increased vascular permeability via its antioxidant, anti-inflammatory effects, and anti-apoptotic effects [3].

Clearly there is much about the physiologic effects of albumin that is not yet well understood [8]. These effects are probably altered in various disease states, particularly those associated with oxidant stress such as sepsis, but whether and how these changes are involved in the pathogenesis of these conditions requires further elucidation. Administration of exogenous albumin may help restore or provide additional antioxidant capacity, transport capabilities, and vascular barrier competence, which may account for some of the beneficial effects of albumin seen in specific patient populations, but it is difficult to differentiate these from albumin’s effects on intravascular volume.

### Hypoalbuminemia

Hypoalbuminemia (generally defined as a serum albumin concentration ≤30 g/l) [5,30] is very common in critically ill patients, the main reasons probably being increased albumin losses from bleeding and via the gastrointestinal tract [31], increased capillary permeability leading to a redistribution from the intravascular to the interstitial space (previously called third-spacing) [32], and dilution from intravenous fluid administration Moreover, in some patients – particularly older patients – baseline albumin levels may already be low as a result of poor nutritional status or altered liver function. Although animal models suggested that albumin synthesis may be reduced in critical illness [33], synthesis appears to be increased in critically ill humans [34].

Importantly, whatever the underlying mechanisms, hypoalbuminemia is associated with worse outcomes including increased complications [5,35-38] and reduced short-term [5,39-43] and longer-term [42,44] survival in critically ill patients. In a meta-analysis of 90 cohort studies that had evaluated hypoalbuminemia as a prognostic biomarker in acutely ill patients, each 10 g/l decrease in serum albumin concentration was associated with a 137% increase in the odds of death, an 89% increase in morbidity, and a 71% increase in length of hospital stay [5]. There is therefore a clear association between the albumin level and the severity of the insult [45], but it remains uncertain whether the effect of hypoalbuminemia on outcome is a cause–effect relationship or whether hypoalbuminemia is rather a marker of serious disease.

### Early clinical trials

#### *For resuscitation in heterogeneous groups of critically ill patients*

Although albumin solutions were first introduced in the 1940s, the first RCT of albumin administration was only published some 30 years later in 1975 (Table 1). This early RCT, conducted in just 16 patients undergoing abdominal aortic surgery, compared the effects of intraoperative use of albumin solution with those of a sodium-rich fluid during surgery and showed that albumin infusion led to less extracellular fluid expansion [46]. Other relatively small studies followed, so that by the time the Cochrane meta-analysis [2] was published in 1998 the average sample size of the 32 included studies was just 46 patients. Although the results of many studies take years to be published and to change clinical practice – if indeed they ever do – this Cochrane report influenced practice rapidly around the world, especially in the UK where use of albumin decreased by 40 to 45% in the 6 months after publication [47]. An Expert Working Party of the Committee on Safety of Medicines in the UK highlighted the thoughts of many in the medical community that there was an urgent need to conduct large multicenter RCTs to determine whether albumin administration did indeed worsen outcomes [48]. In 2004, the results of the Saline versus Albumin Fluid Evaluation (SAFE) RCT in almost 7,000 critically ill patients were published, showing that a 4% albumin solution was as safe as normal saline when used as a resuscitation fluid [49].

**Table 1:** Key points in the albumin story so far

**Year**	**Event**	**Reference**
1941	First clinical use of human albumin solution in a patient with multiple trauma and circulatory shock	[7]
1943	One of the first published reports of human albumin use in 200 patients	[50]
1975	First randomized controlled trial of human albumin in 16 patients undergoing abdominal aortic surgery	[46]
1998	Cochrane meta-analysis including 30 randomized controlled trials and reporting increased mortality rates in critically ill patients who received albumin	[2]
1998	US Food and Drug Administration issued a ‘Dear Doctor’ letter to all healthcare providers expressing serious concern over the safety of albumin administration in the critically ill population, based on the findings of the Cochrane meta-analysis, and urging physicians to exercise discretion in its use	[51]
1999	Expert Working Party of the Committee on Safety of Medicines in UK concluded that there was insufficient evidence of harm to warrant withdrawal of albumin products but large, purpose-designed, randomized, controlled clinical trials should be conducted to answer questions about mortality effects	[48]
1999	Study in 126 patients with cirrhosis and spontaneous bacterial peritonitis randomized to treatment with intravenous cefotaxime or cefotaxime and intravenous albumin; hospital and 3-month mortality rates were lower in the patients who received albumin	[52]
2001	Wilkes and Navickis’ meta-analysis including 55 trials and reporting no overall effect of albumin on mortality	[53]
2003	Meta-analysis of 90 cohort studies evaluating hypoalbuminemia as an outcome predictor by multivariate analysis and nine prospective controlled trials evaluating use of albumin to correct hypoalbuminemia; results showed hypoalbuminemia to be a dose-dependent predictor of poor outcome and correction of serum albumin to >30 g/l associated with reduced complications	[5]
2004	Large SAFE study randomizing 6,997 patients to 4% albumin or normal saline when fluid challenge needed; results showed no difference in mortality rates among groups, and subgroup analyses suggested benefit in patients with severe sepsis and harm in those with traumatic brain injury	[49,54,55]
2005	US Food and Drug Administration issued a notice stating that the SAFE study had resolved the prior safety concerns raised by the Cochrane Injuries Group in 1998	[56]
2005	Results of SOAP observational study showing that albumin use was associated with decreased mortality in critically ill patients using a Cox proportional hazard model and a propensity case-matching analysis	[57]
2006	Pilot study of 100 patients with serum albumin ≤30 g/l randomized to receive 300 ml of 20% albumin solution on the first day and then 200 ml/day if their serum albumin concentration remained <31 g/l, or to receive no albumin; organ function was improved in patients treated with albumin	[30]
2011	Meta-analysis including 17 studies in patients with sepsis reporting a survival benefit for patients who received albumin	[58]
2012	ESICM taskforce Consensus statement suggesting that albumin may be included in the resuscitation of severe sepsis patients (grade 2B)	[59]
2013	Surviving Sepsis Campaign guidelines for the first time specifically suggest (grade 2C) use of albumin in the fluid resuscitation of severe sepsis and septic shock when patients require substantial amounts of crystalloids	[60]
2013	EARSS randomized controlled multicenter study comparing 100 ml 20% albumin with normal saline in patients with early severe sepsis, showing no differences in mortality rates between groups	[61]
2014	ALBIOS randomized controlled multicenter study comparing 20% albumin plus crystalloid or crystalloid alone and then continuing albumin infusions to maintain serum albumin ≥30 g/l; no overall difference in 28-day or 90-day mortality rates but survival benefit at 90 days in patients with septic shock	[62]

#### *For resuscitation in patients with sepsis*

Subgroup analysis of the SAFE study suggested there may be a benefit in patients with severe sepsis (35% of whom had septic shock), with an adjusted odds ratio for death of 0.71 (95% CI, 0.52 to 0.97; *P* = 0.03) for albumin compared with saline [54]. A subsequent meta-analysis that included 17 RCTs comparing albumin solutions with other fluids for fluid resuscitation in patients with sepsis reported that albumin use was associated with decreased mortality (odds ratio, 0.82; 95% CI, 0.67 to 1.0; *P* = 0.047) [58]. Guidelines currently suggest (grade 2C) that albumin use should be considered as a resuscitation fluid in patients with severe sepsis, particularly if those patients are not responding to crystalloid infusion [59,60], based on data from the meta-analysis [58] and preliminary data from a multicenter study in France that suggested a nonsignificant reduction in mortality in patients with septic shock who received albumin [61].

#### *For resuscitation in patients with traumatic brain injury*

In the SAFE trial, patients with traumatic brain injury treated with albumin had worse outcomes than saline-treated patients [55]. Using pattern mixture modeling, the probable mechanism for the increased mortality appeared to be albumin-induced increases in intracranial pressure [63]. The hypotonic and hypooncotic nature of the albumin solution used may also have played a role [64].

#### *For albumin replacement in patients with hypoalbuminemia*

The effects of increasing albumin concentrations by giving exogenous albumin have also been investigated in the critically ill. A meta-analysis of nine prospective controlled trials on correcting hypoalbuminemia in acutely ill patients suggested that complication rates were reduced in patients who achieved serum albumin concentrations >30 g/l after albumin administration [5]. However, in a subgroup analysis of the SAFE study in patients with hypoalbuminemia, using a cutoff value of 25 g/l [4], there were no significant differences in outcomes in hypoalbuminemic patients and normoalbuminemic patients who received albumin. In a pilot RCT of 100 hypoalbuminemic critically ill patients who were randomized either to receive 300 ml of 20% albumin solution on the first day and then 200 ml/day if the serum albumin concentration remained <30 g/dl or to receive no albumin, Dubois and colleagues reported that organ function (as assessed by change in the Sequential Organ Failure Assessment score) improved more in the albumin-treated patients (*P* = 0.03) [30]; these patients also had a less positive fluid balance (*P* =0.04). There was also a beneficial effect on cumulative calorie intake during the first week, suggesting that albumin may have helped decrease intestinal edema.

The effects of albumin administration may also depend on the simultaneous use of diuretics to prevent an albumin infusion-induced increase in hydrostatic pressure, which may increase (rather than decrease) edema formation. Some studies have suggested that the concurrent use of albumin may increase furosemide-induced diuresis in hypooncotic patients with acute respiratory distress syndrome/acute lung injury [65,66] and cirrhosis-induced ascites [67], although not in all critically ill patients [68]; whether this strategy has any effect on patient-centered clinical outcomes is unclear.

In 1999 Sort and colleagues published the results of a RCT in 126 patients with cirrhosis and spontaneous bacterial peritonitis comparing treatment with intravenous cefotaxime or cefotaxime plus intravenous albumin for plasma volume expansion [52]. Renal impairment developed in fewer patients in the patients who received albumin (*P* = 0.002) and these patients also had reduced hospital and 3-month mortality rates (both *P* = 0.01). A more recent RCT reported beneficial effects of albumin plus antibiotic on renal and circulatory function in 110 patients with cirrhosis and infections other than spontaneous bacterial peritonitis; treatment with albumin was an independent predictive factor of survival [69]. A meta-analysis of 16 RCTs also suggested that albumin use was associated with a significant reduction in mortality (odds ratio, 0.46; 95% CI, 0.25 to 0.86) and renal impairment (odds ratio, 0.34; 95% CI, 0.15 to 0.75) in patients with cirrhosis and any infection [70]. Two small RCTs have also demonstrated improved renal function in patients with cirrhosis and hepatorenal syndrome treated with albumin and terlipressin [71,72].

### Recent randomized controlled trial results

Following the results of the SAFE study suggesting a benefit of albumin administration in patients with sepsis, several groups designed RCTs to further evaluate albumin use in this specific group of patients.

In the ALBIOS study, conducted in 100 ICUs in Italy [62], 1,818 patients with severe sepsis or septic shock were randomized either to receive 300 ml of 20% albumin plus crystalloid or to receive crystalloid alone initially to achieve the target resuscitation goals of the early goal-directed therapy protocol used by Rivers and colleagues [73]. Over the subsequent 28 days, albumin infusions were adjusted to maintain serum albumin ≥30 g/l; crystalloid solutions were given when considered clinically indicated by the attending physician. More patients in the albumin group than in the crystalloid group reached the target mean arterial pressure within 6 hours after randomization (86% versus 82.5%, *P* = 0.04), and during the first 7 days the mean arterial pressure was higher and the net fluid balance lower in the albumin group than in the crystalloid group [62], despite similar amounts of fluid being administered to the two groups. There were, however, no overall differences in 28-day mortality rates (32% albumin vs 32% crystalloid; relative risk in the albumin group, 1.00; 95% CI, 0.87 to 1.14; *P* = 0.94) or 90-day mortality rates (41% albumin vs 44% crystalloid; relative risk, 0.94; 95% CI, 0.85 to 1.05; *P* = 0.29) between the groups. Of the 1,818 patients, 579 (31.8%) were randomized within 6 hours and 1,239 (68.2%) more than 6 hours after meeting the clinical criteria for severe sepsis; there were no significant differences in outcomes according to the interval between meeting clinical criteria and randomization. In the subgroup of patients with septic shock at enrollment (*n* = 1,121), however, those who received albumin had significantly lower 90-day mortality rates than those who received saline (44% versus 50%; relative risk, 0.87; 95% CI, 0.77 to 0.99; *P* = 0.03) [62].

In the multicenter EARSS study in France, so far published only in abstract form [61], 798 patients with septic shock of less than 6 hours duration were randomized to receive 100 ml of 20% albumin or 100 ml of 0.9% saline every 8 hours for 3 days. Almost all patients had severe hypoalbuminemia at study inclusion. There were no significant differences in mortality rates between the two groups (24.1 vs 26.3%).

## Where next?

Having briefly reviewed the background to the albumin story so far, where are we left? Who, if anyone, should be given albumin? Some answers will be provided from further analysis of the results from recent and ongoing studies, but in the meantime we believe there are six key questions that need answering.

### Resuscitation versus supplementation (medication)?

Initially considered largely as an acute resuscitation fluid for its beneficial short-term effects on COP and blood volume, recognition of the adverse outcomes associated with hypoalbuminemia and new knowledge about vascular barrier functioning has led to an increased interest in use of albumin solutions as a supplement to correct and maintain albumin levels. Nevertheless, it is difficult to separate volume effects from the effects of maintenance of serum albumin – particularly in critically ill patients, many of whom are hypoalbuminemic and in whom it is difficult to clearly relate the timing of interventions to the onset of disease. Thus, most studies of albumin administration actually combine a degree of resuscitation with a degree of supplementation/maintenance of serum albumin. As a resuscitation fluid, the major benefit of albumin will be from its impact on COP, resulting in a short-term increase in intravascular volume. As supplementation, effects on COP are also important, potentially reducing the risk of interstitial edema, but some of albumin’s other actions, such as transport and antioxidant effects, may also become important.

Efforts to substitute synthetic colloids for albumin as part of perioperative fluid therapy have not been very successful. Hydroxyethylstarch solutions can persist for long durations in the skin, the liver and, most importantly, the kidney [74], with a potential risk of renal failure and even increased mortality rates in septic patients [75]. Gelatin solutions have been less well studied, in part because they are not available in the United States, and their persistence is quite short.

### Which concentration of albumin solution?

Albumin solutions are available in a variety of concentrations, mainly 20 to 25% or 4 to 5%, and which concentration should be used has generated some debate. Direct blood volume measurements in humans have revealed that the intravascular volume effect of isooncotic preparations of human albumin solutions is much higher than that of crystalloids (>80% vs <20%) [76]. However, the albumin concentration chosen largely depends on whether other fluids, especially crystalloids, are administered simultaneously – a 20% (20 g in 100 ml) albumin solution given simultaneously with 500 ml of normal saline solution is equivalent to a 3.3% (20/600) albumin solution. Nevertheless, hyperoncotic albumin may be a better choice if edema is already present [77], avoiding excessive sodium and chloride loads and their attendant complications [78].

### Which dose of albumin?

Determining the ideal dose or volume of albumin that should be used is difficult. Early physiological studies demonstrated that administration of 5% albumin to septic patients expanded the plasma volume by an amount equivalent to the volume infused [79]. Different studies have used different doses, and perhaps the dose should be adjusted according to a target serum albumin concentration, as in the ALBIOS study [62]. The dose chosen by Mira and colleagues in the EARSS study (100 ml of 20% albumin 8 hourly for 3 days) achieved an increase in serum albumin to 25 to 29 g/l [61], raising the possibility that the albumin dose used may have been too low to show definite benefit – although this increase was similar to that reported in the SAFE study subgroup analysis of patients with sepsis, in whom a benefit was reported [54].

### Should albumin infusions target albumin levels?

The need to make decisions as to whether or not a particular patient should receive albumin based on their albumin level is related to whether the considered use is targeted as resuscitation or supplementation. Most patients requiring resuscitation fluids in the ICU are hypoalbuminemic and, as mentioned earlier, the fluid will be given largely for its effects on COP – that is, limiting edema formation – provided that the hydrostatic pressure does not increase excessively. In such patients, monitoring the albumin concentration is probably of little value.

In more prolonged administration as supplementation, however, serum albumin levels may be a useful guide to ongoing needs, in combination with disease severity, hemodynamic status, and nutritional status; just as an arbitrary cutoff hemoglobin concentration should not be used to define absolute need for blood transfusion in all patients, so a specific serum albumin threshold for albumin administration is unlikely to be relevant to all. The meta-analysis of nine prospective controlled trials on correcting hypoalbuminemia in acutely ill patients mentioned earlier suggested that complication rates were reduced in patients who achieved serum albumin concentrations >30 g/l after albumin administration [5]. As a result, the ALBIOS study protocol stipulated that albumin administration should be titrated to maintain serum albumin ≥30 g/l [62]. Albumin levels were measured on a daily basis and 200 or 300 ml of 20% albumin were administered in patients with albumin levels between 25 and 30 g/l or <25 g/l, respectively. Following the time course of albumin levels, especially in response to an albumin infusion, may be more valuable than a single albumin level, but optimal albumin levels during critical illness are not clearly defined.

### What type of albumin preparation?

Currently available human albumin solutions are developed using various techniques, such that the various commercially available albumin solutions may differ in protein content and composition, binding capacity, metal ion content, antioxidant properties, charge, capacity to bind drugs, and so forth [80,81]. This is a difficult topic to evaluate because there are few data available about the precise composition of the different albumin solutions and whether or how this may impact on its clinical properties [81,82], but the differences may help explain some of the different study results. One study comparing six different commercially available preparations of albumin with serum albumin from healthy volunteers reported large differences between the solutions, particularly in terms of the presence of oxidized cysteine 34 (23% in human volunteer albumin vs 54 to 60% in commercial preparations) [81], which may influence the properties and hence the clinical impact of albumin solutions [82]. Precise compositions of albumin solution should be clearly identified in future study reports.

### Is albumin cost-effective?

Albumin solutions have a good safety record [83]. The large SAFE study reported that 4% albumin was as safe as normal saline in a heterogeneous group of ICU patients [49] and meta-analyses have noted that albumin has a better safety profile than other colloid solutions [84,85]. Reports of adverse events, including anticoagulant and allergic effects and transmission of microorganisms, are rare. In a study evaluating adverse event reporting between 1998 and 2000, the incidence of all reported serious nonfatal and fatal adverse events was just 5 per million doses, and no patient death was classified as probably related to albumin administration [83].

Albumin is, however, more costly than all other resuscitation fluids, although prices have decreased relative to other fluids over the last 10 years. Nevertheless, if shown to reduce morbidity and mortality even by a small amount, it is likely that the cost-effectiveness ratio would favor albumin because so many ICU interventions are very costly. There have been very few cost-effectiveness evaluations of albumin use in the ICU. Guidet and colleagues assessed the cost-effectiveness of albumin as given in the SAFE study on patients with severe sepsis and septic shock admitted to one of 35 French ICUs [86]. Based on a presumed 4.6% reduction in mortality associated with albumin therapy (as shown in the SAFE study), 513 lives were saved among the 11,137 patients included, with an estimated life expectancy for each life saved of 9.8 years. The cost per life saved was estimated at €6,037 and the cost per life-year saved as €617. The authors therefore concluded that albumin administration was a cost-effective intervention in patients with severe sepsis or septic shock [86]. Most recently, a cost-effectiveness analysis in severe sepsis and septic shock using an advanced Bayesian approach observed life-years gained with albumin relative to crystalloid therapy, and concluded that albumin may be the most cost-effective intravenous solution in this patient population [87].

## Recommendations and conclusions

Many changes within intensive care medicine have come as the result of the realization that traditional practices once thought to be therapeutic were in fact detrimental [88]. Human albumin solutions have been available for almost 60 years and provide effective resuscitation with less fluid required than for crystalloid solutions; for many years, however, albumin was widely and perhaps nondiscriminantly used as a resuscitation fluid with little realization that it may not be appropriate in all patients. Although criticized for its methodology and the heterogeneity of the studies included, the 1998 Cochrane meta-analysis brought this possibility to the attention of the wider community and stimulated the conduct of large, multicenter RCTs [2]. As the results of these studies become available, the role of albumin in today’s critical care unit is becoming clearer and several recommendations can be made:

• Albumin administration, although unlikely to cause harm in most patients, is not necessary in all critically ill patients and should be reserved for use in specific groups of patients in whom there is evidence of benefit.

• A hypotonic albumin solution should be avoided as a resuscitation fluid in patients with traumatic brain injury, based on the results of the SAFE subgroup analysis [55].

• There is now enough evidence – albeit largely from subgroup analyses – and plausible biological rationale to support use of albumin in patients with septic shock when a colloid is considered [54,62].

• Albumin administration should be considered in patients with cirrhosis and spontaneous bacterial peritonitis [52], but possibly also other infections [69,70]; in hypooncotic patients with acute respiratory distress syndrome [65,66]; and also in patients with cirrhosis and type 1 hepatorenal syndrome [89].

Future research should be focused on patients who are most likely to benefit from albumin administration in whom the evidence is inadequate or controversial because of conflicting study results. In addition to observational cohort studies and RCTs focusing on specific patient groups, mechanistic studies are necessary to further elucidate the molecular and physiologic rationale for the beneficial effects of albumin and explore how albumin’s pleiotropic actions may be important in specific groups of critically ill patients. Such studies also need to better elucidate the mechanisms of development of hypoalbuminemia. Studies also need to clarify issues of dosage and appropriate targets and whether different albumin solutions have unique differential effects on patients’ responses and outcomes to albumin administration. The effects of albumin infusions on drug (especially antimicrobial) dosing also need clarification.

## Abbreviations

COP: Colloid osmotic pressure; RCT: Randomized controlled trial; SAFE: Saline versus Albumin Fluid Evaluation.

## Competing interests

J-LV has no conflicts of interest related to this article. JAR reports patents owned by the University of British Columbia that are related to the genetics of sepsis and its treatment; the University of British Columbia has also submitted a patent related to the use of vasopressin in septic shock. JAR is an inventor on these patents. JAR also reports receiving consulting fees from Ferring Pharmaceuticals (which manufactures vasopressin and is developing selepressin), Grifols (which sells albumin), Trius Pharmaceuticals (which is developing antibiotics), and Sirius Genomics Inc.; and reports having received grant support from Sirius Genomics, Ferring Pharmaceuticals, and Astra Zeneca that is provided to and administered by the University of British Columbia. MJ has held lectures for B. Braun, Fresenius Kabi, Serumwerk Bernburg, Baxter and Grifols; has received unrestricted research grants from Fresenius Kabi, CSL Behring, Serumwerk Bernburg and Grifols; and is a member of the Grifols AAB. GM serves on advisory boards for Grifols and CSL Behring, and his institution has received research grant support from Baxter Healthcare. BG has received honoraria for lectures from Fresenius Kabi and LFB, and he is on the advisory board of Grifols and Fresenius Kabi. JW is a member of the Advisory Boards and an invited speaker for Baxter, Danone, Fresenius-Kabi, Grifols, and Nestlé. RFR has received honoraria for lectures from Pfizer, Astellas and Grupo Ferrer, and is on the Advisory boards of Grifols, Bellco and Grupo Ferrer. SAM has received speaker's honoraria from Fresenius Kabi. LG is on the Advisory board of Grifols and has received speaker’s honoraria from KCI, B. Braun, Baxter, Grifols, and Kedrion.
