# Erratum to: Albumin administration in the acutely ill: what is new and where next?

**DOI:** 10.1186/s13054-014-0630-x

**Published:** 2014-11-19

**Authors:** Jean-Louis Vincent, James A Russell, Matthias Jacob, Greg Martin, Bertrand Guidet, Jan Wernerman, Ricard Ferrer, Stuart A McCluskey, Luciano Gattinoni

**Affiliations:** Department of Intensive Care, Erasme Hospital, Université libre de Bruxelles, route de Lennik 808, 1070 Brussels, Belgium; Center for Heart Lung Innovation and Critical Care Medicine, University of British Columbia and St. Paul’s Hospital, Vancouver, V6Z BC Canada; Department of Anaesthesiology, University Hospital Munich, Nussbaumstraße 20, 80336 Munich, Germany; Department of Medicine, Division of Pulmonary, Allergy and Critical Care, Emory University School of Medicine, Atlanta, GA 30303 USA; Service Réanimation Médicale, Hôpital Saint Antoine, Universitaires Est Parisien, Paris, France; Sorbonne Universités, UPMC Université Paris 06, UMR-S 1136, Institut Pierre Louis d’Epidémiologie et de Santé Publique, Paris, F-75013 France; Department of Anesthesiology & Intensive Care Medicine, Karolinska University Hospital, Huddinge, 14186 Stockholm, Sweden; Servei de Medicina Intensiva, Hospital Universitari Mútua Terrassa, Universitat de Barcelona, CIBER Enfermedades Respiratorias, Terrassa, 08221 Barcelona, Spain; Department of Anesthesia and Pain Management, Toronto General Hospital, University of Toronto, Toronto, ON M5G 2C4 Canada; Dipartimento di Anestesia, Rianimazione (Intensiva e Subintensiva) e Terapia del Dolore, Fondazione IRCCS Ca’ Granda – Ospedale Maggiore Policlinico, Milan, 20122 Italy

## Erratum

After publication of this review [1], we noticed that the author name Ricard Ferrer Roca should instead be stated as Ricard Ferrer.
